# Long-Term Anatomical Durability and Clinical Outcomes of Concomitant Laparoscopic Sleeve Gastrectomy and Hiatal Hernia Repair: Up to 10-Year Multicenter Analysis

**DOI:** 10.1007/s11695-026-08803-1

**Published:** 2026-06-20

**Authors:** Francesco Maria Carrano, Beatrice De Luca, Francesco Angrisani, Giulia Griguolo, Fabio Cesare Campanile, Nicola Perrotta, Cristian Eugeniu Boru, Luigi Angrisani, Gianfranco Silecchia

**Affiliations:** 1 Department of Medical and Surgical Sciences and Translational Medicine, Faculty of Medicine and Psychology, Sant’ Andrea Hospital, Sapienza University of Rome, Rome, Italy; 2 Sapienza University of Rome, Rome, Italy; 3 Department of Medicine, University of Naples Federico II, Naples, Italy; 4 Department of General Surgery, Ospedale di Civita Castellana, Civita Castellana, Italy; 5 Ospedale San Carlo, Potenza, Italy; 6 Department of Public Health, University of Naples Federico II, Naples, Italy

**Keywords:** Sleeve gastrectomy, Hiatal hernia repair, Bariatric surgery, Gastroesophageal reflux disease, Long-term outcomes, Recurrence

## Abstract

**Background:**

Hiatal hernia is common in patients with severe obesity undergoing metabolic bariatric surgery. Although concomitant hiatal hernia repair during laparoscopic sleeve gastrectomy has been widely adopted, long-term data beyond 5 years remain limited, and the clinical relevance of anatomical recurrence is still poorly defined.

**Objectives:**

To evaluate the 10-year recurrence rate, predictors of failure, and clinical outcomes following concomitant sleeve gastrectomy and hiatal hernia repair.

**Methods:**

This retrospective multicenter cohort study analyzed 111 patients who underwent sleeve gastrectomy with concomitant hiatal hernia repair between 2014 and 2015. Complete-case analysis included only patients with assessable recurrence status at follow-up. The primary outcome was anatomical recurrence (> 2 cm transhiatal migration of the gastric sleeve). Secondary outcomes included reintervention, gastroesophageal reflux disease (GERD), and weight loss.

**Results:**

Among 111 patients, 108 were analyzed. Mean age was 50.1 ± 11.6 years, 80.6% female, mean baseline BMI 42.7 ± 5.8 kg/m², and mean follow-up was 93.0 ± 22.7 months (7.7 ± 1.9 years). Hernia recurrence occurred in 14 patients (13.0%). Among these, 5 (35.7%) underwent reintervention, while 9 (64.3%) were managed conservatively. Freedom from recurrence was 98.1% at 5 years and 68.9% at 10 years, although the 10-year estimate should be interpreted cautiously. Hernia size ≥ 5 cm and postoperative GERD were associated with recurrence (OR 5.0; 95% CI 1.2-20.0; P=0.035; OR 4.97; 95% CI 1.56–17.53; *p* = 0.008). Reintervention was required in 11 patients (10.2%), all with symptomatic GERD.

**Conclusions:**

Concomitant sleeve gastrectomy and hiatal hernia repair were associated with acceptable long-term outcomes in this multicenter cohort. Reintervention was more often associated with postoperative reflux-related symptoms than with anatomical recurrence alone, while many anatomical recurrences were managed conservatively.

## Introduction

Hiatal hernia is highly prevalent in patients with obesity, affecting approximately 37%-39.8% of those undergoing bariatric surgery [1, 2]. This high prevalence is attributed to elevated intra-abdominal pressure, increased body mass index (BMI), and anatomical factors that contribute to progressive weakening of the diaphragmatic crura and phreno-esophageal ligament [3, 4]. The concurrent presence of hiatal hernia and/or gastroesophageal reflux disease (GERD) represents a significant clinical challenge in the management of patients undergoing primary laparoscopic sleeve gastrectomy.

Sleeve gastrectomy has become as the most performed bariatric procedure worldwide, accounting for 61% of all primary bariatric operations globally [5, 6]. While sleeve gastrectomy provides excellent weight loss and metabolic outcomes, concerns remain regarding its effect on GERD, since it may exacerbate pre-existing reflux or induce de novo reflux through several mechanisms, including blunting of the angle of His, disruption of sling fibers, increased intragastric pressure, and reduced gastric compliance [7–9]. Reported GERD rates after sleeve gastrectomy vary widely from 24% to 59%, reflecting heterogeneity in diagnostic criteria, follow-up duration, and patient selection [10, 11].

The management of concurrent hiatal hernia during sleeve gastrectomy remains controversial within the bariatric surgery community. Some surgeons advocate routine concomitant hiatal hernia repair to restore anatomical integrity and potentially reduce reflux risk [12, 13], whereas others question the value of repairing small defects, citing limited evidence of long-term benefit [14, 15]. Proponents of concomitant hiatal hernia repair argue that restoration of the diaphragmatic hiatus and reconstruction of the phreno-esophageal ligament may preserve lower esophageal sphincter competence and minimize reflux [16]. However, the impact of hiatal hernia repair on long-term anatomical durability, GERD outcomes, and reintervention rates remains incompletely studied, particularly beyond 5-year follow-up.

Recent meta-analyses have reported conflicting results regarding the efficacy of sleeve gastrectomy with hiatal hernia repair compared with sleeve gastrectomy alone. Some studies have shown improvement in GERD [12, 17–19], whereas others have found no significant difference [15, 20]. A major limitation of the current literature is the scarcity of long-term data beyond 3–5 years, the lack of standardized GERD diagnostic criteria, and the absence of routine objective assessments with pH monitoring or manometry [21]. Furthermore, the natural history of anatomical recurrence, the optimal management of recurrent hernia, and the predictors of anatomical failure remain poorly defined.

Despite the widespread adoption of concomitant hiatal hernia repair during laparoscopic sleeve gastrectomy, long-term data beyond 5 years remain scarce, and the clinical relevance of anatomical recurrence is poorly defined. In particular, it remains unclear whether postoperative gastroesophageal reflux is a consequence of anatomical failure or simply a parallel marker of adverse long-term outcomes. We hypothesized that postoperative GERD, rather than anatomical recurrence alone, may be more closely associated with long-term clinical failure and reintervention after sleeve gastrectomy with concomitant hiatal hernia repair.

## Methods

This retrospective multicenter cohort study included patients who underwent laparoscopic sleeve gastrectomy with concomitant hiatal hernia repair at four Italian bariatric centers between January 2014 and December 2015. The study was conducted in accordance with the Declaration of Helsinki and was approved by the institutional review board (Protocol No. 1880/2025). All patients provided written informed consent for the procedure and for the collection of their data.

Inclusion criteria were age 18–65 years, BMI ≥ 35 kg/m² with obesity-related comorbidities or ≥ 40 kg/m², documented hiatal hernia diagnosed preoperatively (upper gastrointestinal endoscopy, barium esophagogram) or intraoperatively, undergoing primary sleeve gastrectomy with concomitant hiatal hernia repair, and a minimum 5-year follow-up with assessable recurrence status.

Exclusion criteria included: prior bariatric or antireflux surgery, severe GERD with Los Angeles grade C/D esophagitis or Barrett’s esophagus, paraesophageal hernias requiring emergent repair, and incomplete follow-up for recurrence assessment.

All patients underwent a comprehensive preoperative evaluation, which included demographic and anthropometric data, as well as a comorbidity assessment. Objective reflux testing with pH-impedance monitoring or esophageal manometry was not routinely performed, reflecting the retrospective real-world nature of the study period. GERD was therefore defined clinically, using patient-reported symptoms, proton pump inhibitor use, and endoscopic findings with a Los Angeles classification of esophagitis when available. Hiatal hernia diagnosis and characterization were established preoperatively by upper endoscopy and/or barium esophagogram, or intraoperatively during systematic hiatal dissection. No patient without either preoperative or intraoperative evidence of hiatal hernia was included.

Hiatal hernia size was measured intraoperatively and classified arbitrarily as large if the transverse (radial) crural defect measured ≥ 5 cm using atraumatic grasper jaws (2.5 cm width) as reference, or small when the defect was < 5 cm.

Surgical details were recorded from operative reports and reflected local center practice rather than a pre-specified study protocol. At the time of LSG, the hiatus was inspected systematically and any true hiatal defect was repaired. When HHR was indicated, the distal esophagus was mobilized to achieve adequate intra-abdominal length before crural closure. Posterior cruroplasty was the standard repair; anterior suturing and mesh reinforcement were used selectively. Biosynthetic mesh (GORE^®^ BIO-A^®^ Tissue Reinforcement, W. L. Gore & Associates, Inc., Flagstaff, AZ, USA) was used to reinforce the hiatoplasty in large hiatal defects or in patients deemed at high risk for recurrence due to poor tissue quality, most commonly weakness of the right crus. The extent of gastric mobilization, bougie size, and staple-line reinforcement were not standardized across centers and were left to the operating surgeon. Patients received routine postoperative care according to each center’s bariatric pathway, including thromboprophylaxis, early mobilization, dietary progression, and short-term proton pump inhibitor therapy. Routine postoperative vitamin supplementation was also prescribed as part of standard bariatric care. An oral water-soluble contrast study was performed on postoperative day 2 before discharge. Follow-up visits were scheduled at 1, 3, 6, 12, and 24 months and then annually. Follow-up assessment included weight, BMI, excess weight loss, GERD symptoms, PPI use, quality-of-life information when available.

As part of standard bariatric follow‑up, patients were scheduled for periodic upper endoscopy at approximately 3‑year intervals, in addition to symptom‑driven endoscopic or radiologic investigations. The primary outcome was anatomical hernia recurrence rate, defined as radiographic or endoscopic evidence of transhiatal migration of the gastric sleeve greater than 2 cm. In patients without contemporaneous long‑term imaging, recurrence status at the last follow‑up visit was clinically inferred using a predefined composite definition. Patients were classified as having no recurrence if they reported no upper gastrointestinal symptoms suggestive of hiatal hernia or reflux, were not receiving PPI therapy for reflux‑related indications, had no prior imaging documenting sleeve migration or hiatal hernia recurrence during follow‑up, without clinically significant weight regain or indication for revisional surgery.

To maximize transparency and minimize misclassification bias, patients with an uncertain recurrence status who failed to meet these criteria were excluded from the complete-case analysis, reflecting the retrospective, practice-based nature of this dataset.

Secondary outcomes included time to recurrence, predictors of hernia recurrence, reintervention rate and indications, GERD prevalence at final follow-up, weight loss outcomes, postoperative complications, and mortality.

### Statistical Analysis

Continuous variables are presented as mean ± standard deviation (SD) or median with interquartile range (IQR) as appropriate. Categorical variables are presented as frequencies and percentages. Complete-case analysis was performed, including only patients with documented recurrence status at final follow-up. An independent samples t-test was used for continuous variables. Chi-square test or Fisher’s exact test was used for categorical variables. Odds ratios (OR) with 95% confidence intervals (CI) were calculated for binary outcomes. The Kaplan-Meier analysis was used to estimate the freedom from recurrence over time. Patients were censored at the time of their last endoscopic or radiographic assessment. Univariate logistic regression identified potential predictors of recurrence. Variables with *p* < 0.10 were entered into a multivariate logistic regression model to identify independent risk factors. Variables analyzed included: age, sex, BMI, hernia size (large vs. all other defects), hernia type, mesh use, suture type, operating time, and postoperative GERD. Statistical significance was defined as *p* < 0.05 (two-tailed). All analyses were performed using R version 4.5.2. 22.

## Results

The study cohort included 111 patients who underwent sleeve gastrectomy with hiatal hernia repair during the study period. Of those, three patients with incomplete documentation of recurrence status were excluded from the primary analysis. Therefore, 108 patients with complete postoperative follow-up data and documented recurrence status were included in the final analysis. Baseline demographic and clinical characteristics are summarized in Table 1. The mean age was 50.1 ± 11.6 years (median, 51.0; range, 25–73 years). The majority were female (87 patients, 80.6%). Mean preoperative BMI was 42.7 ± 5.8 kg/m², ranging from 30 to 58 kg/m². Baseline comorbidities were highly prevalent, reflecting the typical metabolic burden of severe obesity: hypertension (36.1%), obstructive sleep apnea (36.1%), non-alcoholic fatty liver disease (30.6.7%), osteoarthritis (27.8%), dyslipidemia (22.1 %), and diabetes mellitus (11.1%).

**Table 1:** Baseline Demographic and Clinical Characteristics

Characteristic	Value
Demographics	
Age (years), mean ± SD	50.1 ± 11.6
Age (years), median (IQR)	51.0 (41.0–60.0)
Age range (years)	25–73
Female sex, n (%)	87 (80.6%)
Male sex, n (%)	21 (19.4%)
Anthropometrics	
Preoperative BMI (kg/m²), mean ± SD	42.7 ± 5.8
BMI range (kg/m²)	30–58
Baseline Comorbidities, n (%)	
Hypertension	39 (36.1%)
Obstructive sleep apnea	39 (36.1%)
Non-alcoholic fatty liver disease	33 (30.6%)
Osteoarthritis	30 (27.8%)
Dyslipidemia	24 (22.2%)
Type 2 diabetes mellitus	12 (11.1%)
Coronary artery disease	2 (1.9%)
Congestive heart failure	1 (0.9%)
Hiatal Hernia Characteristics	
Sliding hernia, n (%)	106 (98.1%)
Type II paraesophageal hernia, n (%)	2 (1.9%)
Non-large hernia (< 5 cm), n (%)	88 (81.5%)
Large hernia (≥ 5 cm), n (%)	11 (10.2%)
Hernia size not measured, n (%)	9 (8.3%)
Operative Characteristics	
Characteristic	Value
Operating time (min), mean ± SD	115.7 ± 43.8
Operating time (min), median (IQR)	105.0 (90.0-125.0)
Mesh reinforcement, n (%)	36 (32.4%)

*BMI* Body mass index, *IQR* Interquartile range, *SD* Standard deviation

Across the cohort, hiatal hernia was diagnosed preoperatively in 85 of 111 patients (76.6%), whereas in 33 patients (29.7%) the defect was identified intraoperatively; in 7 cases (6.3%) the preoperative diagnosis was confirmed intraoperatively. Hiatal hernias were primarily of the sliding type (98.2%, *n* = 106), with only two patients (1.8%) having type II paraesophageal hernias. Regarding defect size, 11 patients (10.2%) presented with large hernias (≥ 5 cm). The remaining 97 patients (89.8%) had small (< 5 cm) or unmeasured defects and were grouped for the analysis. Symptomatic esophagitis Los Angeles grade A–B was present in 55 patients (49.5%).

Mean operative time was 115.8 ± 44.7 minutes (median 105 min, range 60–340 min). Hiatal hernia repair technique was consistent across centers. Mesh reinforcement was used selectively in 36 patients (32.4%), primarily for large hiatal defects. No conversions to open surgery occurred, and there were no intraoperative complications requiring deviation from the planned procedure. Early postoperative complications were rare. Only one major complication (Clavien-Dindo grade ≥ II) occurred (0.9%). This patient developed a gastric perforation two years after the index operation, requiring gastrectomy with esophago-jejunal anastomosis. Rehospitalization within 30 days occurred in 17 patients (15.3%), mainly for nausea, vomiting, or dehydration that were managed conservatively. There were no perioperative deaths (0% mortality).

Mean follow-up duration was 93.0 ± 22.7 months (7.7 ± 1.9 years), with a median of 90 months (IQR: 74–111 months). Follow-up ranged from 60 to 144 months (5 to 12 years). Follow-up imaging (upper endoscopy and/or esophagogram) was performed in 59 patients (53.2%) at a median of 12–18 months postoperatively or when clinically indicated for symptoms.

**Fig. 1:**
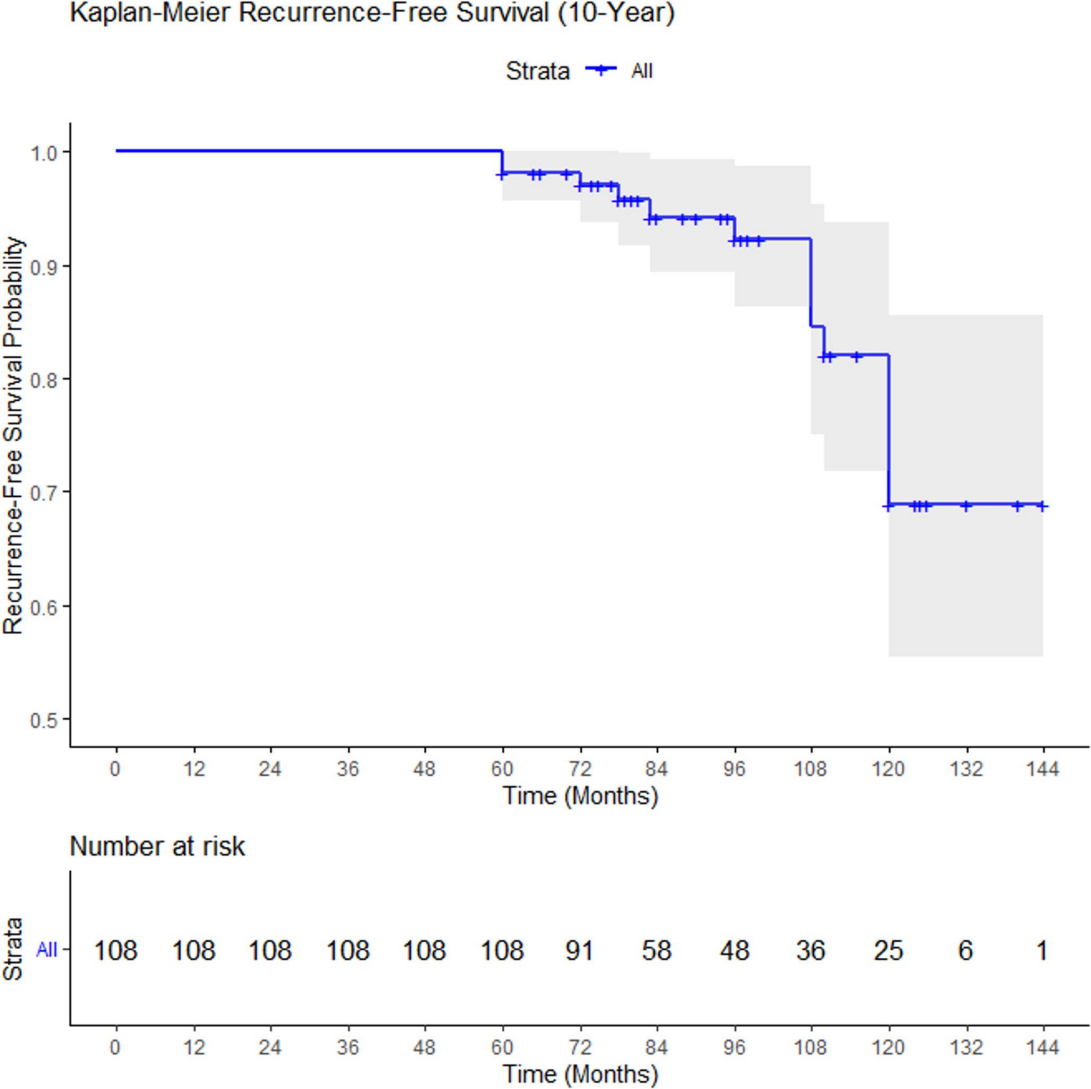
Kaplan–Meier estimates of freedom from recurrence over time. The Kaplan-Meier curve demonstrates the probability of remaining free from anatomical hiatal hernia recurrence over the 10-year follow-up period. The estimates of freedom from recurrence rate were 98.1% at 5 years, and 68.9% at 10 years. Shaded regions represent the 95% confidence interval. Numbers at risk are displayed below the x-axis. Median estimates of freedom from recurrence not reached (> 50% remain recurrence-free at 10 years)

Among 108 evaluable patients, hernia recurrence was documented in 14 patients (13.0%; 95% CI: 7.9%–20.6%) (Table 2). The Kaplan-Meier analysis (Fig. 1) suggested a decline in freedom from recurrence over time, from 98.1% at 5 years to 68.9% at 10 years. However, the Kaplan–Meier estimates of freedom from recurrence declined to 68.9% at 10 years (95% CI: 54.3%–83.5%). This apparent late decrease reflected recurrences occurring within the relatively small subgroup of patients with complete decennial follow-up, with only 25 patients remaining at risk at 120 months and should therefore be interpreted with caution.

**Table 2:** Primary outcome: hernia recurrence

Outcome	*N* = 108	%	95% CI
Recurrence Status			
Hernia recurrence	14	13.0%	7.9%-20.6%
No recurrence	94	87.0%	79.4%-92.1%
Management of Recurrence (*n* = 14)			
Surgical reintervention	5	35.7%	
Conservative management	9	64.3%	
Temporal distribution (*n* = 14)			
Early (< 12 months)	2	14.3%	
Intermediate (1–7 years)	6	42.9%	
Late (> 7 years)	6	42.9%	
Time to Recurrence			
Mean ± SD (months)	78.8 ± 38.5		
Median (IQR) (months)	80.5 (60.9-109.5)		
Range (months)	2.2–120.0		

*CI* Confidence interval, *IQR* Interquartile range, *SD* Standard deviation

Management of recurrence was largely conservative: only 5 of the 14 patients (35.7%) required reoperation, while the remaining 9 were successfully treated with medical therapy alone (Table 2). When analyzing the total burden of reoperations (*n* = 11) (Table 3), symptomatic GERD was the leading indication (*n* = 6), exceeding anatomical recurrence (*n* = 5). Specifically, the 6 patients with refractory GERD were converted to Roux-en-Y gastric bypass RYGB (*n* = 3) or one anastomosis gastric bypass (OAGB) (*n* = 3; at other institutions). The 5 patients operated for recurrence underwent conversion to RYGB (*n* = 3), OAGB (*n* = 1), or anterior cruroplasty with mesh reinforcement (*n* = 1). This confirms that intractable symptomatic GERD, rather than anatomical failure, was the primary driver of surgical revision.

**Table 3:** Reintervention Rates and Indications

Reintervention Indication	*N*	% of Total Cohort	% of Reinterventions
Total Reinterventions	11	10.2%	100%
Symptomatic GERD/esophagitis	6	5.6%	54.5%
Hiatal Hernia recurrence	5	4.6%	45.5%
Type of Reintervention			
Conversion to RYGB	6	5.6%	54.5%
Conversion to OAGB	4	3.7%	36.4%
Anterior cruroplasty with mesh	1	0.9%	9.1%
Management of Recurrence (*n* = 14)			
Surgically treated	5	35.7% of recurrences	
Conservatively managed	9	64.3% of recurrences	

*GERD *Gastroesophageal reflux disease, *OAGB* One-anastomosis gastric bypass, *RYGB* Roux- en-Y gastric bypass

Univariate analysis identified significant predictors of hernia recurrence (Table 4). Patients with large hernias (≥ 5 cm) experienced a recurrence rate of 36.4% (4/11), compared to 10.3% (10/97) in all other defects (OR 5.0, 95% CI: 1.2–20.0, *p* = 0.035). Postoperative GERD emerged as the strongest independent predictor, with recurrences occurring in 26.5% (9/34) of symptomatic patients versus only 6.8% (5/74) of those without GERD. This corresponds to a nearly 5-fold increase in risk (OR 4.97, 95% CI: 1.6–16.3, *p* = 0.01), a finding further supported by multivariate analysis adjusted for BMI and age, confirming a strong independent association without implying direct causality (Table 5). While non-sliding hernia types showed a trend toward higher recurrence (*p* = 0.0098), the statistical power of this observation was limited by the small sample size, as only two patients presented with paraesophageal components.

**Table 4:** Univariate analysis: predictors of hernia recurrence

Variable	Recurrence (*n* = 14)	No Recurrence (*n* = 94)	*P*-value	Odds Ratio (95% CI)
Large hernia (≥ 5 cm)	4 (28.6%)	7 (7.4%)	0.035	5.0 (1.2–20.0)
Postoperative GERD	9 (64.3%)	25 (26.6%)	0.011	4.97 (1.56–17.53)
Mesh use	6 (42.9%)	27 (28.7%)	0.353	1.9 (0.6–5.9)
Age (years)	50.3 ± 10.3	50.0 ± 11.8	0.917	—
BMI (kg/m²)	43.9 ± 7.1	42.6 ± 5.6	0.506	—
Operating time (min)	106.2 ± 17.2	117.7 ± 48.3	0.159	—

*BMI *Body mass index, *CI *Confidence interval, *GERD *Gastroesophageal reflux disease, *SD *Standard deviation

**Table 5:** Multivariate analysis of factors associated with hernia recurrence

Variable	aOR	95% CI	*p*-value
Large hernia (≥ 5 cm)	6.41	1.2–20	0.027
Postoperative GERD	6.37	1.6–16.3	0.006
Mesh use	1.88	0.6–5.9	0.359
BMI (per unit)	1.00	0.9–1.1	0.996
Age (per year)	0.99	0.9–1.1	0.796

*aOR* Adjusted odds ratios, *CI* Confidence interval, *BMI* Body mass index

**Fig. 2:**
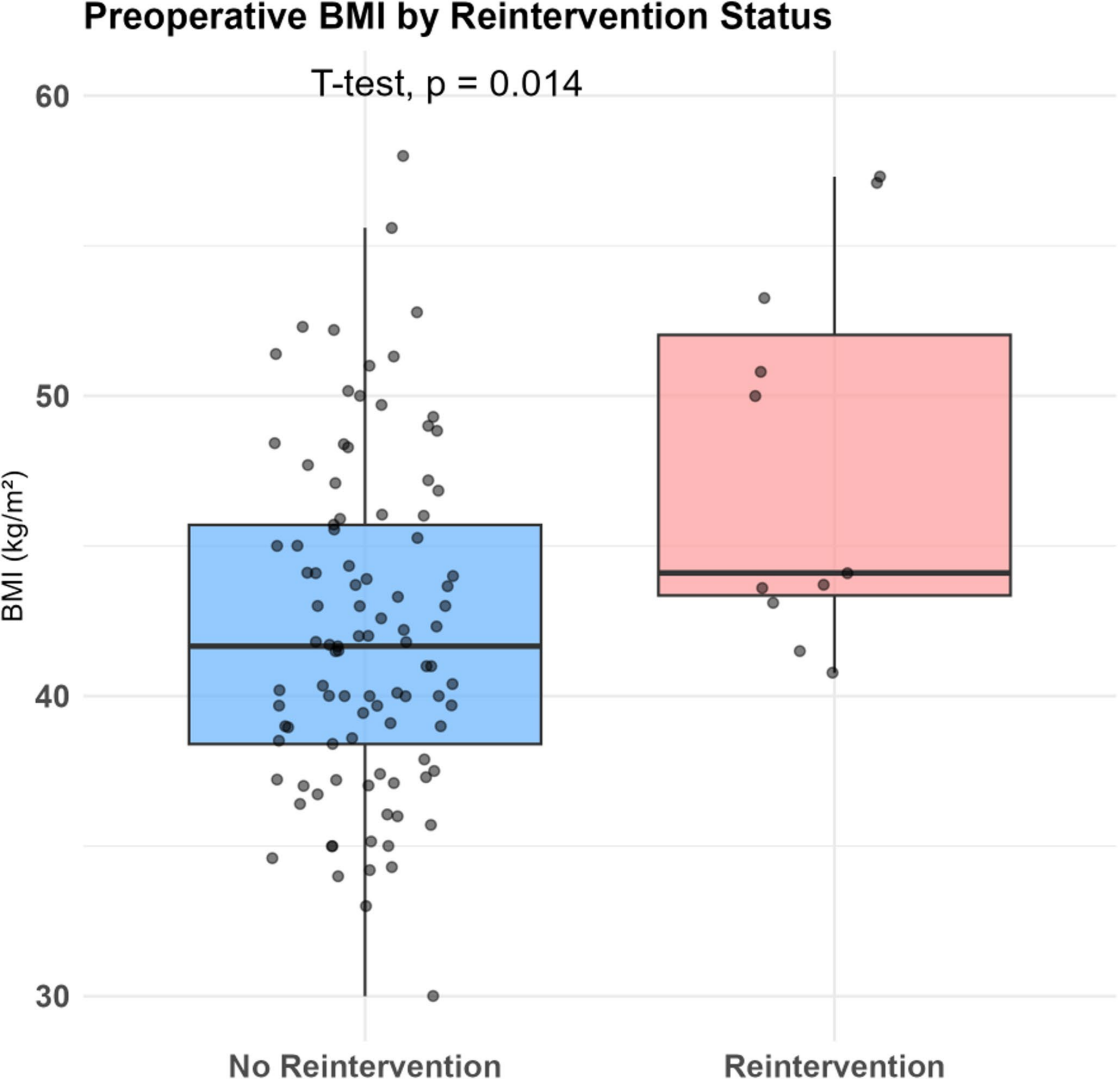
Preoperative BMI by Reintervention Status

**Fig. 3:**
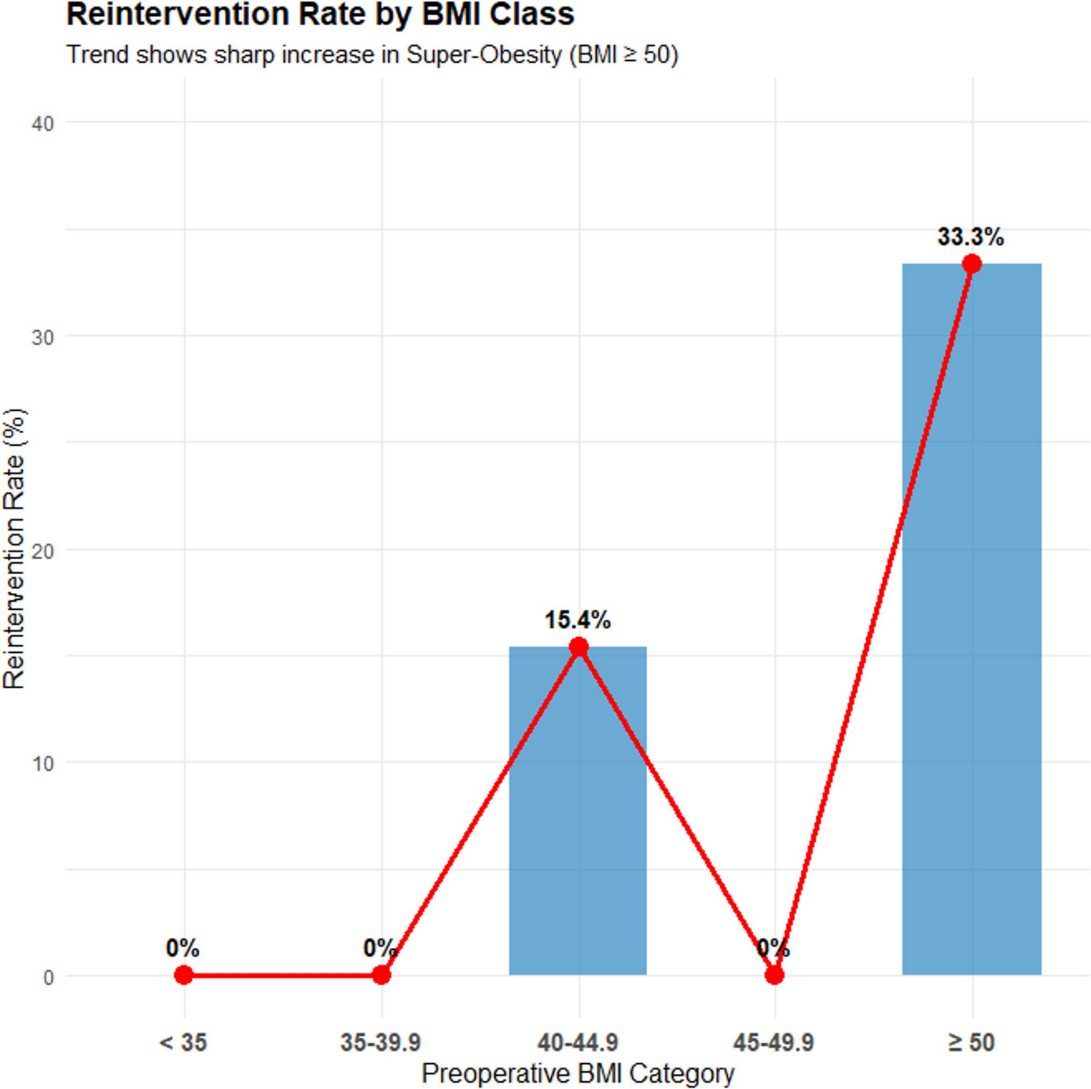
Surgical reintervention rates stratified by preoperative BMI class. The chart illustrates the percentage of patients requiring reintervention for reflux or recurrence within each BMI category. The red trend line highlights a non-linear risk profile, with a sharp escalation in risk observed in patients with BMI ≥ 50 kg/m², who had a reintervention rate of 33.3%. In contrast, patients with a BMI < 40 kg/m² had a 0% reintervention rate in this cohort. The association between higher BMI and reintervention risk was statistically significant (*p* = 0.014)

Mesh reinforcement was used selectively in larger (hernia size ≥ 5 cm) and more complex defects. In this context, no statistically significant association between mesh use and recurrence was observed (OR 1.86, 95% CI 0.59–5.87, *p* = 0.353), although the crude recurrence rate was numerically higher in the mesh group than in the non-mesh group (18.2% vs. 10.7%). Preoperative BMI was significantly higher in patients requiring reintervention (47.7 vs. 42.2 kg/m², *p* = 0.014) (Fig. 2). Stratification by BMI class revealed that patients with BMI ≥ 50 kg/m² had a reintervention rate of 33.3%, significantly higher than lower BMI groups (Fig. 3).

At final follow-up, symptomatic GERD was present in 34 patients (31.5%). An additional three patients (2.8%) were asymptomatic but required ongoing PPI therapy for gastric protection due to concomitant medications, bringing the total GERD-related treatment burden to 37 patients (34.3%). While 65.7% of patients were completely free of reflux symptoms and medication, approximately one-third required some form of GERD management, highlighting GERD as a common concern after sleeve gastrectomy even with concomitant hiatal hernia repair.

Among the 14 patients with hernia recurrence, nine (64.3%) had postoperative GERD. Conversely, among the 34 patients with symptomatic postoperative GERD, nine (26.5%) developed recurrence. This significant association (*p* = 0.01) supports a close relationship between reflux-related symptoms and anatomical recurrence; however, in this retrospective cohort, the temporal sequence and causal direction of this association cannot be reliably determined.

BMI changes from baseline to final follow-up demonstrated excellent weight loss maintenance, with a decrease from a preoperative mean of 42.7 ± 5.8 kg/m² to a postoperative mean of 29.1 ± 4.6 kg/m². This corresponds to a BMI reduction of 13.6 ± 5.3 kg/m² (31.4% ± 10.1% reduction), with no significant weight regain observed during the 10-year follow-up, indicating durable bariatric outcomes despite the concomitant hiatal hernia repair.

## Discussion

This study provides one of the longest available follow-up analyses of concomitant hiatal hernia repair during laparoscopic sleeve gastrectomy and suggests that postoperative GERD, rather than anatomical recurrence alone, was more strongly associated with long-term clinical failure in this cohort. These findings challenge a purely anatomy-driven interpretation that equates hiatal hernia recurrence with surgical failure. Instead, our data suggest that postoperative reflux-related symptoms may better capture clinically meaningful outcomes and the likelihood of revisional surgery. However, the temporal and causal relationship between GERD and anatomical recurrence cannot be established in this retrospective study. Our principal findings are: (1) acceptable long-term anatomical durability with a 13% recurrence rate at 10 years, (2) postoperative GERD showed the strongest association with recurrence in our analysis (approximately fivefold higher odds), and (3) reflux-related symptoms were the leading clinical indication for reintervention, either in isolation or together with HH recurrence, (4) conservative management is feasible for the majority of recurrences (64.3%), (5) sustained weight loss is maintained through 10 years without compromise from concomitant hiatal hernia repair, and (6) the concomitant repair appears safe without additional intra or perioperative complication even when crural reinforcement was used.

Recent meta-analyses report pooled recurrence rates ranging from 2% to 11% [17, 22] with significant heterogeneity attributed to variations in follow-up duration, diagnostic criteria, and surgical technique. Other contemporary evidence reports on the effectiveness of sleeve gastrectomy with concomitant hiatal hernia repair [23]. A key finding of our study is the divergence between the crude recurrence rate (13%) and the 10-year Kaplan–Meier estimates of freedom from recurrence over time (69%). This discrepancy highlights the limitations of reporting simple percentages in bariatric cohorts with variable follow-up. The temporal distribution of recurrences, with 42.9% occurring after 7 years, suggests that anatomical failure after concomitant HHR may occur late in a subset of patients. However, late Kaplan–Meier estimates should be interpreted with caution, as only 25 patients remained at risk at 120 months, and the corresponding confidence intervals were wide. Therefore, the apparent decline observed at 10 years should not be overinterpreted.

One of the most clinically relevant findings of our study is the strong association between postoperative GERD and adverse long-term clinical outcomes, including both anatomical recurrence (OR 4.97; 95% CI 1.56-17.53; p=0.011, *p*= 0.008) and surgical reintervention. In our series, all patients requiring reoperation reported symptomatic GERD. These findings suggest that reflux-related symptoms may represent an important marker of clinically meaningful failure, beyond anatomical recurrence alone, although the direction of this association remains uncertain. The absence of routine objective reflux testing precludes differentiation between true GERD, functional symptoms, non-acid reflux, and motility-related complaints. Accordingly, postoperative GERD in the present study should be interpreted as a clinically defined reflux-related outcome rather than a physiologically confirmed diagnosis according to Lyon criteria.

Several mechanisms may explain the strong association between GERD and recurrence:

1. Increased intragastric pressure from reflux episodes may lead to gastric acids exposure and chronic stress on crural repair, impairing tissue healing and fibrosis at the crural repair site, weakening long-term structural integrity and predisposing to gradual separation [9, 24]
2. Delayed gastric emptying associated with GERD may increase fundic distension, promoting cranial migration of the gastroesophageal junction [24, 25]
3. Disruption of the His Angle and resection of the sling fibers in the distal part of the lower sphincter, which results in low esophageal-sphincter pressure [9, 24]
4. Common risk factors, such as tissue quality, hiatal dimensions, and sphincter dysfunction, may predispose individuals to both GERD and recurrence independently [3, 26]

Notably, the prevalence of symptomatic GERD in our cohort was 31.5%. While this data aligns with reported rates following sleeve gastrectomy alone (20–40%) [11, 27], it is important to contextualize this finding within our high-risk population, where all our patients had a confirmed hiatal hernia at baseline. In such a population, sleeve gastrectomy alone would have been associated with significantly higher rates of reflux and anatomical failure. Therefore, concomitant hiatal hernia repair is observed to be associated with a mitigated reflux burden in this baseline cohort, maintaining the GERD rate within the range of standard sleeve gastrectomy outcomes despite the adverse anatomical baseline.

Large hiatal hernias (≥ 5 cm) were associated with a 5-fold increased recurrence risk (36.4% vs. 10.3%, *p* = 0.035), consistent with published literature reporting recurrence rates of 15–29% for large defects [28–30]. The heightened failure rate likely reflects:

1. Greater tissue tension required for crural approximation in large defects, predisposing to suture pull-through or tissue necrosis [31]
2. Intrinsically poor tissue quality in obese patients with chronic hernia stretching, limiting capacity for durable repair [32, 33]
3. Higher baseline GERD prevalence in large hernias, introducing confounding through the GERD-recurrence association [34]

Mesh reinforcement was used selectively, mainly in patients with larger or higher-risk defects. No statistically significant association between mesh use and recurrence was identified (OR 1.86, 95% CI 0.59–5.87, *p* = 0.353), although the crude recurrence rate was numerically higher in the mesh group than in the non-mesh group (18.2% vs. 10.7%). This finding should be interpreted cautiously because of likely confounding by indication, and no causal inference can be drawn regarding the effect of mesh reinforcement on recurrence risk in the present study [35–37].

A novel contribution of our study is the detailed categorization of reintervention indications, which suggests that reflux-related symptoms, rather than anatomical recurrence alone, were more often associated with the need for reintervention in this cohort. Among 11 reinterventions, 6 (54.5%) were conducted for refractory GERD alone, and 5 (45.5%) for both hernia recurrence and GERD. Furthermore, among 14 anatomical recurrences, only 5 (35.7%) required surgery, while 9 (64.3%) were managed conservatively with PPI therapy and lifestyle modification. The latter finding challenges the assumption that all recurrences require reoperation and suggests a more tailored approach to post-recurrence management. Conversion to bypass procedures was the predominant reintervention strategy (90%), aligning with established evidence that bypass procedures provide superior GERD resolution compared to other revisional sleeve procedures [35, 38]. Meta-analyses report 60–95% GERD remission following sleeve gastrectomy conversion to RYGB [36, 37], making bypass procedures the preferred option for refractory reflux. Our finding that 64.3% of recurrences were managed conservatively, with reasonable symptomatic control, has important clinical implications. This suggests that anatomical recurrence does not necessarily mandate reoperation, and that many patients can tolerate small recurrent hernias without significant morbidity. Factors favoring conservative management include:

- Small recurrence size (<3 cm)
- Minimal or absent symptoms
- Good response to PPI therapy
- Absence of complications (incarceration, volvulus, obstruction)

This approach aligns with contemporary hernia surgery principles, emphasizing symptom-driven rather than anatomy-driven management [39, 40]. Importantly, no patient managed conservatively in our series required emergent surgery, confirming the safety of this approach.

In terms of weight control, the mean BMI reduction of 13.6 kg/m² (31.4% reduction) with sustained maintenance through 10 years demonstrates that concomitant hiatal hernia repair does not compromise bariatric efficacy [19, 41]. This finding refutes concerns that hiatal dissection and crural repair may impair gastric emptying or caloric restriction mechanisms.

Our findings should be contextualized within the broader landscape of sleeve gastrectomy variants designed to mitigate GERD risk. For example, a recent meta-analysis report superior GERD control for sleeve gastrectomy with fundoplication compared to sleeve gastrectomy with hiatal hernia repair alone (5% vs. 20%, *p* < 0.001) [20]. However, sleeve-fundoplication carries higher complication rates (perforation: 3%, mortality: 0.5%) [20] and longer-term data are lacking. Preliminary reports on sleeve gastrectomy with Hill repair or anterior fundoplication suggest promising GERD outcomes [42, 43], but these techniques remain investigational with limited multicenter validation.

Primary RYGB for GERD patients remains the most established revisional option for obese patients with established GERD, offering remission rates of 60–80% [44]. However, patient and surgeon preference for laparoscopic sleeve gastrectomy technical simplicity and revisability often drives the selection of sleeve-based approaches. Nevertheless, conversion to RYGB remains the standard of care also for patients requiring reoperation due to persistent or de novo GERD. This is particularly true for lean patients who have achieved weight loss goals but suffer from reflux, where RYGB demonstrates excellent outcomes as a salvage procedure.

This study has several limitations. First, the primary analysis was based on a complete-case approach, including only patients with available recurrence status at follow-up and excluding those without sufficient data for recurrence assessment. This introduces a potential risk of selection bias, as patients with assessable long-term recurrence status may have differed systematically from those without adequate follow-up data. Patients who remained under closer surveillance because of symptoms or clinical concern were more likely to undergo diagnostic evaluation and, therefore, to have recurrence detected, potentially inflating the observed recurrence rate among evaluable cases. Conversely, patients with incomplete follow-up may have had asymptomatic or otherwise undocumented recurrences that remained unrecognized, potentially leading to underestimation of the true recurrence burden. Accordingly, neither the direction nor the magnitude of this bias can be established with certainty. Second, in a substantial proportion of patients, recurrence status was inferred from available clinical follow-up rather than confirmed by protocolized imaging. This may have resulted in underdetection of asymptomatic recurrences and, consequently, may have affected the reported recurrence estimates. This limitation is inherent to the retrospective real-world design of the study, in which endoscopic and radiographic assessments were generally performed selectively, mainly in symptomatic patients, rather than contemporaneous imaging, despite a follow‑up pathway including periodic 3‑year endoscopy and symptom‑triggered investigations. This real‑world pattern likely underestimates asymptomatic anatomical recurrences and makes the observed 13% recurrence rate a conservative estimate of the true failure burden, a common occurrence in long-term retrospective cohorts. Third, objective reflux testing with pH-impedance monitoring and manometry was not routinely available; therefore, GERD assessment reflected retrospective clinical follow-up rather than a protocolized diagnostic pathway. As a result, postoperative GERD in the present study should be interpreted as a clinically defined reflux-related outcome rather than as a physiologically confirmed diagnosis according to Lyon criteria. Fourth, although the overall operative strategy was shared across centers, some technical details were not fully standardized, including the type of crural closure, the selective use of mesh reinforcement, and staple-line management. This procedural heterogeneity reflects real-world multicenter practice but may have introduced additional variability in both anatomical and clinical outcomes, thereby limiting causal inference regarding the impact of individual technical factors. Finally, the relatively small number of recurrence events limits the robustness of predictor analysis. In particular, associations identified in regression models should be interpreted cautiously, as the limited event count reduces estimate stability and increases the risk of imprecision.

These limitations are balanced by the multicenter design and the uniquely long follow-up duration, which contribute meaningful data on 10‑year outcomes after concomitant sleeve gastrectomy and hiatal hernia repair, an area with limited existing evidence.

Future research should prioritize trials that utilize objective pH monitoring to define optimal repair techniques and mesh indications. Furthermore, studies validating safety criteria for conservative management of recurrence and developing preoperative algorithms to stratify patients between concomitant repair and primary gastric bypass are essential.

## Conclusions

Concomitant LSG and HHR were associated with acceptable long-term outcomes in this multicenter cohort. Reintervention was more often associated with postoperative reflux-related symptoms than with anatomical recurrence alone, while many anatomical recurrences were managed conservatively. These findings should be interpreted cautiously considering the retrospective design, incomplete objective follow-up, and limited number of recurrence events.

## Data Availability

De-identified patient data are available upon reasonable request to the corresponding author, subject to institutional review board approval and data sharing agreements.
